# Improving occupational health care for construction workers: a process evaluation

**DOI:** 10.1186/1471-2458-13-218

**Published:** 2013-03-11

**Authors:** Julitta S Boschman, Henk F van der Molen, Judith K Sluiter, Monique HW Frings-Dresen

**Affiliations:** 1 Academic Medical Center, University of Amsterdam, Department: Coronel Institute of Occupational Health, PO Box 22660, Amsterdam, 1100, DE, the Netherlands; 2 Arbouw, Dutch Health & Safety Institute in the Construction Industry, Harderwijk, The Netherlands

## Abstract

**Background:**

To evaluate the process of a job-specific workers’ health surveillance (WHS) in improving occupational health care for construction workers.

**Methods:**

From January to July 2012 were 899 bricklayers and supervisors invited for the job-specific WHS at three locations of one occupational health service throughout the Netherlands. The intervention aimed at detecting signs of work-related health problems, reduced work capacity and/or reduced work functioning. Measurements were obtained using a recruitment record and questionnaires at baseline and follow-up. The process evaluation included the following: reach (attendance rate), intervention dose delivered (provision of written recommendations and follow-up appointments), intervention dose received (intention to follow-up on advice directly after WHS and remembrance of advice three months later), and fidelity (protocol adherence). The workers scored their increase in knowledge from 0–10 with regard to health status and work ability, their satisfaction with the intervention and the perceived (future) effect of such an intervention. Program implementation was defined as the mean score of reach, fidelity, and intervention dose delivered and received.

**Results:**

Reach was 9% (77 workers participated), fidelity was 67%, the intervention dose delivered was 92 and 63%, and the intervention dose received was 68 and 49%. The total programme implementation was 58%. The increases in knowledge regarding the health status and work ability of the workers after the WHS were graded as 7.0 and 5.9, respectively. The satisfaction of the workers with the entire intervention was graded as 7.5. The perceived (future) effects on health status were graded as 6.3, and the effects on work ability were graded with a 5.2. The economic recession affected the workers as well as the occupational health service that enacted the implementation.

**Conclusions:**

Programme implementation was acceptable. Low reach, limited protocol adherence and modest engagement of the workers with respect to the intervention were the most prominent aspects that influenced the intervention process. The increase in the workers’ knowledge about their health status and work ability was substantial, and the workers’ satisfaction with the intervention was good. The perceived effect of the advised preventive actions on health status was sufficient.

**Trial registration:**

Netherlands Trial Register: http://NTR3012

## Background

Construction is a large, dynamic, and complex sector, creating employment for millions of people worldwide. Unfortunately, it is also the sector with the most fatal on-the-job injuries and high numbers of nonfatal occupational injuries and illnesses involving days away from work [1]. During the last decade, efforts have been focused on improving occupational safety and health throughout the world [2]. One strategy to improve the health of construction workers, is to offer them a periodical workers’ health surveillance programme (WHS) [3]. Such a programme is aimed at detecting pre-clinical and clinical abnormalities indicating work-related diseases and changes in health. If necessary, preventive actions can be taken to prevent deterioration of the workers’ health status and improve work functioning [3].

WHS for construction workers should serve multiple purposes that are consistent with the guidelines for WHS as stated by the International Labour Organisation (ILO) [3]. First, WHS allows an evaluation of the effectiveness of control measures in the workplace, such as controlling dust [4] or noise [5], by measuring the related health effects, such as respiratory problems [6] or noise-induced hearing loss [7]. Secondly, adverse health effects, such as musculoskeletal disorders [8], mental complaints [9] or skin disorders [10], can be detected at an early stage when interventions are likely to be beneficial, thereby preventing further deterioration of workers’ health. Third, safe methods of work and health maintenance can be reinforced using counselling with an occupational physician (OP). Fourth, WHS that focuses on a specific occupation allows for an assessment of the work ability for that particular type of work and evaluation of whether and to what extent the workplace should be adapted to the worker (Boschman JS, van der Molen HF, Sluiter JK, Frings-Dresen MHW. Evaluating physical work ability in construction workers: a multiple case study, Submitted).

In the Netherlands, employees in the construction industry are offered the opportunity to participate in WHS programme once every four years (when they are under the age of 40) or every two years (when they are over 40 years old). The Health and Safety Institute for the Dutch construction industry provides this service for all employees in the construction industry. Employees are invited by an occupational health service (OHS) near their place of residence and subsequently they can decide whether they will voluntarily attend their WHS. WHS is believed to have preventive effects, but principally this outcome depends on the quality and appropriateness of the instruments used in screening for adverse health effects or reduced work functioning [3]. Moreover, screening can only have a preventive effect when adequate actions are taken in accordance with the signs detected; in other words, both the OP and the workers should take action when warranted. For example, the OP can arrange a workplace visit to address risk factors at the worksite, while the worker himself can undertake personal actions such as visiting a physical therapist or better utilise his hearing protection. Thus, the preventive effect of WHS depends largely on the behaviour of both occupational health professionals and workers regarding the preventive actions after the initial WHS itself.

To contribute to improvement in the quality of occupational health care for construction workers, we developed WHS in which both the quality and adequacy of the screening instruments and the potentially most effective interventions are attuned to a specific occupation [11]. We have chosen to develop and evaluate such a job-specific WHS for two specific and very distinct construction occupations: one as an example of a physically demanding construction occupation (bricklayers) and one as an example of a mentally demanding construction occupation (construction supervisors).

Regarding the question of whether this job-specific WHS is effective, there is a growing tendency to evaluate the process involved. Failures in this process might affect the outcome obtained with the job-specific WHS [12]. The focus of a process evaluation is not on testing differences between control and intervention group, but on the process of an intervention to increase the understanding of factors possibly affecting outcomes of an intervention. A thorough assessment of the process is essential for assessing the internal and external validity of the job-specific WHS. In the review of Durlak and Dupre [12] it was found that at least 23 contextual factors might influence implementation. Therefore, in a process evaluation multiple variables are studied based on several data sources. Process evaluations have been used to provide a detailed insight into the implementation of, for example, a lifestyle programme for construction workers who are at risk for cardiovascular disease [13], a worksite intervention aimed at promoting work ability [14] and a programme for suicide prevention in the construction industry [15]. The aim of the present study is to evaluate the process of implementing job-specific WHS in occupational health care for construction workers in two different occupations in the Netherlands.

## Methods

This process evaluation concerned a study on the effect of job-specific WHS for Dutch bricklayers and construction supervisors. The methods and background of this evaluation of the job-specific WHS have previously been reported in detail [11]. The medical ethics committee of the Academic Medical Center approved the study and the board of the participating OHS provided positive feedback on the local feasibility of the study. All participants gave their written informed consent.

### Study design

The study was designed as a nonrandomised controlled trial involving one OHS with multiple sites in the Netherlands. At three widespread sites of the OHS throughout the Netherlands, bricklayers and supervisors were invited for the job-specific WHS.

### Study population

A total of 899 bricklayers and supervisors were approached for participation from January until July 2012: 1) workers who were eligible for their WHS in the second trimester of 2012 and 2) workers who did not respond to the invitation for their regular WHS in 2011. To restrict the travelling distance, we invited only workers who resided in the region of the OHS’s site at which the job-specific WHS was conducted. All bricklayers and construction supervisors were 1) primarily bricklayer or construction supervisor; 2) male; 3) able to read, speak and write Dutch sufficiently well and 4) not planning to leave their occupation due to resignation or (early) retirement.

The intervention was conducted by three OPs, three ergonomists and a total of five medical assistants. At each site of the OHS, a team of an OP, ergonomist and one or two medical assistants conducted the intervention.

### Procedure

The OHS approached the workers and invited them to participate in the study, conform the standard operating procedure for inviting workers for WHS within the Dutch construction industry. The job-specific WHS was designed for the study and was offered as a ‘special and temporary offer’ to the workers. Workers received an invitation and leaflet with information on the job-specific WHS and the study. If interested, the workers could apply for attendance by returning a letter to the OHS or they could respond by telephone. To hinder occupational care as little as possible, the workers were given the opportunity to attend the job-specific WHS without participating in the evaluation study. When not interested in the job-specific WHS, they were offered the opportunity to participate in the regular WHS, if desired, at another site of the OHS. After attending their job-specific WHS, the workers were asked to participate in the study and complete the informed consent and baseline questionnaire. When they did so, they received an incentive (the bricklayers received an ergonomic trowel, and the supervisors received a safety flashlight). Within two to three months after they had attended their job-specific WHS, the participants received a follow-up questionnaire and incentive (a lottery ticket for a national lottery) at their home address. When the workers’ did not complete or returned their baseline questionnaire (for example because their OP had forgotten to give it to them), we sent information on the study and a questionnaire containing the applicable items of the baseline questionnaire and the follow-up questionnaire two months after the workers had attended their job-specific WHS. Workers who did not return the questionnaire, received a reminder within three weeks.

### Intervention

**Figure 1:**
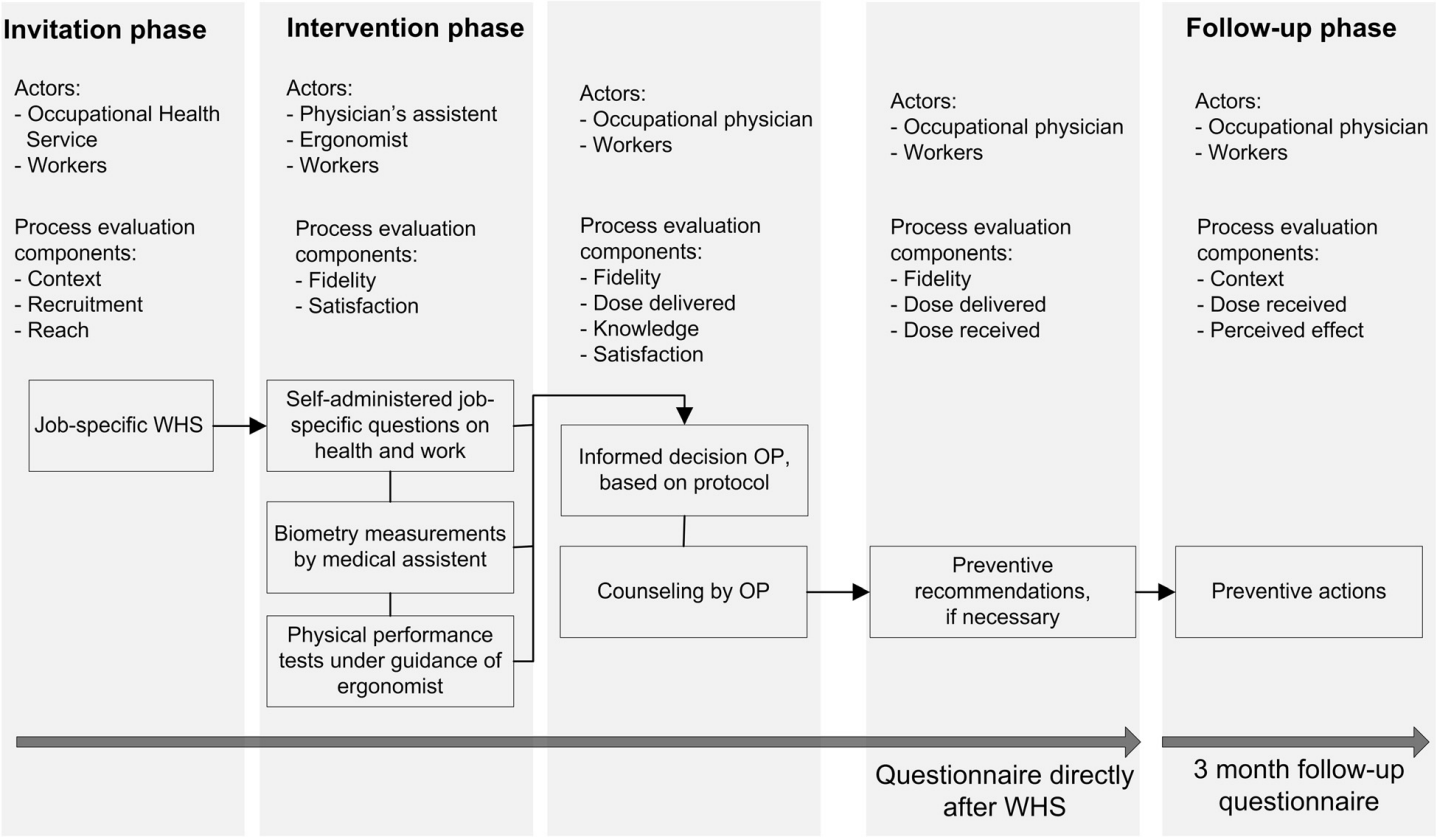
Study phases, actors, content of the intervention and time points of measurement.

The job-specific WHS was aimed at detecting signs of work-related health problems, reduced work capacity and/or reduced work functioning. The following domains are represented: musculoskeletal system, safety (vision, perception of sound, psychological vigilance and working at heights), hazardous substances (skin, lungs), health in relation to work (cardiovascular health) and work ability. Each worker filled in job-specific questions consisting of validated and reliable screening instruments as well as job-specific questions regarding complaints or work limitations, formulated by the research team, to be answered with a yes or no, when no valid instruments were available (‘signalling questions’) [16]. Then biometry measurements were performed by a medical assistant, and subsequently the worker performed physical performance tests under the guidance of an ergonomist. Further details on the content of the job-specific WHS were previously published [11]. The OP used a structured protocol to assess all results and prepared the worker consult. Thereafter, the OP discussed the results and, if necessary, recommended desirable preventive actions to the worker during a 20-min consultation. A structured intervention protocol for the OP facilitated job-specific intervention measures. This intervention protocol for the OP was intended as guidance and allowed the OP to tailor his recommendation to the needs of the individual worker conform the principles of shared decision-making [17]. The worker received a report with the advice of the OP. The OPs and three ergonomists who conducted the intervention participated in a half-day training course provided by an instructor of the Netherlands School of Public & Occupational Health (NSPOH) and the first author. The medical assistants were instructed individually on the protocol by the first author. The differences between the intervention and the usual WHS were as follows: the job-specific content of the self-administered questionnaire, the addition of physical performance tests and the guidance for the OP (Figure 1).

### Measures and data collection procedures

The process evaluation involved multiple measures (Table 1), aimed at gaining insight into the process of the evaluation study.

1. A recruitment record was prepared by the OHS. This record contained information about the workers who were approached, those who applied for attendance, those who attended and the reasons given for nonparticipation;

2. The workers completed questionnaires;

3. The OHS’ registry in which the OPs fill in the intended and finished follow-up appointments with the workers;

4. Within one week after the study period had ended, the participating occupational health professionals (including the OPs, medical assistants, ergonomists and the involved employee of the planning office) were visited by the first author for a semi-structured interview.

**Table 1:** The process evaluation components, description and data sources of the process evaluation of starting job-specific workers’ health surveillance for construction workers

**Component**	**Description**	**Data source**
**Reach**	Participation: the attendance rate	Recruitment record
**Dose received**	Engagement of the worker with the intervention.	Workers’ questionnaires directly after WHS and three months after WHS
	Questions (yes/no):	
	1. Did the OP advise you any preventive actions? (directly after WHS)	
	2. Do you think you will act upon the advice of the OP? (directly after WHS)	
	3. Did the OP advise you any preventive actions? (three months after WHS)	
	4. Did you act upon the advice of the OP? (three months after WHS)	
	Remembrance of the advice at follow-up: agreement between question 1 and 3.	
**Fidelity**	Extent to which the intervention was conducted as designed and the requested protocol was followed, based on eight performance indicators:	Documents of the WHS, checklists and workers’ questionnaire
	1. Correct processing of the questionnaire data by the medical assistant and the OP (yes/no).	
	2. Correct processing of the summary of results of all components of WHS (yes/no).	
	3. Correct processing of the results of the physical performance test by the ergonomist (yes/no).	
	4. Advise to the OP based on the physical performance test by the ergonomist (yes/no).	
	5. Signs to intervene were correctly determined by the OP (yes/no).	
	6. Signs to intervene were prioritised before counselling by the OP (yes/no).	
	7. Evaluation of prioritisation after counselling by the OP (yes/no).	
	8. Written advice provided by the OP.	
	Question (yes/no): Did you receive the written advice of the OP?	
**Dose delivered**	Effort of the OP: Provided the OP their written advice to the worker and accomplished the preventive actions that they intended to initiate?	Workers’ questionnaire, OHS’ registry
**Knowledge**	Increase in the insight of the workers in their health status and work ability (0–10)	Workers’ questionnaire
**Satisfaction**	Satisfaction of the workers with the WHS as a whole and its components (questionnaire, physical performance test, counselling by the OP). (0–10)	Workers’ questionnaire
**Perceived effect**	Perceived (future) effect of the preventive action(s) on health and work ability. (0–10)	Workers’ questionnaire
**Context**	Environment	Interviews with occupational health professionals
	Organisational and financial aspects	
	Individual circumstances	

### Process evaluation components

Based on the process elements as described by Steckler and Linnan [18], the extent to which the intervention was delivered as planned, the exposure and the engagement of the workers with the intervention and the workers’ attitude towards the intervention were included. The process evaluation components are described in more detail below and in Table 2. We operationalised the programme implementation as the mean score of reach, dose delivered, dose received, and fidelity as suggested by Linnan and Steckler [18]. A priori, we estimated a programme implementation of approximately 60% to be acceptable [12].

**Table 2:** Fidelity: score on the individual performance indicators as part of the process evaluation of job-specific workers’ health surveillance for construction workers

**Performance indicator**	**Description**	**Sufficient performance % (relative frequency)**
**1**	Correct processing of the questionnaire data by the medical assistant and OP	16% (12/77)
**2**	Correct processing of the summary of results of all components of the WHS by the OP	58% (45/77)
**3**	Correct processing of the results of the physical performance test by the ergonomist	91% (70/77)
**4**	Advice was given to the OP by the ergonomist based on the physical performance test	73% (56/77)
**5**	Signs to intervene were correctly determined by the OP	16% (12/77)
**6**	Signs to intervene were prioritised before counselling by the OP	95% (73/77)
**7**	Evaluation of the prioritisation after counselling by the OP	97% (75/77)
**8**	Written advice was provided to the worker by the OP	92% (65/71)
	Average total score	67%

### Reach and intervention dose received

The extent to which the workers were exposed to the intervention was operationalised by measuring the reach and intervention dose received. Reach was defined as the attendance rate. Intervention dose received was operationalised by multiple variables: whether the workers had the intention to act upon the advice of the OP directly after the WHS (yes/no) and whether the workers had remembered this advice at the follow-up (yes/no).

### Intervention dose delivered and fidelity

The extent to which the activities of the intervention were executed as planned was measured by the intervention dose delivered and fidelity. The dose delivered was operationalised by multiple variables: whether the OP provided his or her written advice to the worker (yes/no) and whether the OP had accomplished the preventive actions that they intended to initiate, such as a workplace visit or a follow-up appointment (yes/no) as indicated in the OHS’ registry. Fidelity was measured by eight performance indicators, which were scored as sufficient or insufficient. The performance of the medical assistant was measured by verifying whether the questionnaire data and the biometry results were all correctly processed (yes/no). The performance of the ergonomist was measured by verifying whether the results of the physical performance test were all correctly processed and whether recommendations in line with the findings during the test, were given to the OP. The performance of the OP was measured by six performance indicators: whether the questionnaire and biometry data were all correctly processed, whether the summary of all results were correctly processed, whether all signs to intervene were correctly determined, whether the signs to intervene were prioritised prior to the worker consult, whether this prioritisation was evaluated after the worker consult and whether the written advice was provided to the worker. A total score was calculated and related to the total number of items, representing the percentage of sufficient scored performance indicators relative to the total number of performance indicators.

### Workers’ knowledge on health and work ability

The knowledge of the workers regarding their own health status and work ability as a result of the intervention was measured by asking the workers to what extent their knowledge on their health status and their own work ability had increased from 0 (no increase in knowledge) to 10 (much more knowledge). The question was asked separately for the intervention as a whole and its individual components (questionnaire, physical performance tests, counselling by the OP). The rates were defined as no increase in knowledge (0), a slight increase in knowledge (>0 and ≤3), a substantial increase (>3 and ≤7.5) or much more knowledge (>7.5).

### Workers’ attitude towards the intervention: satisfaction and perceived (future) effect

Satisfaction was measured by asking the workers how they rated their satisfaction with the intervention as a whole and it’s individual components (questionnaire, physical performance tests, counselling by the OP) on a scale from 0 (not satisfied) to 10 (very satisfied). The perceived (future) effect of the intervention was measured by asking the workers how they rated the (future) effect of the preventive actions they had undertaken on their health status and work ability (0–10, 0 = no effect, 10 = large effect). The rates were defined as no effect / not satisfied (0), poor (>0 and ≤3), limited (>3 and <6), sufficient (≥6 and <7.5) or good (≥7.5).

### Facilitators and barriers in conducting the intervention and the context

To gain in-depth information on facilitators and barriers in conducting the intervention and the context, the occupational health professionals were interviewed. They were asked about the facilitators and barriers affecting their ability to conduct the intervention. The following topics were addressed: circumstances that might have affected the process of conducting the intervention, the satisfaction of the workers and the knowledge the workers gained from their job-specific WHS. Furthermore, potentially important contextual factors relating to the social, political and economic environment were discussed with, the occupational health professionals, with the sector’s Health and Safety Institute and among the research team.

### Analysis

All quantitative data were analysed using descriptive statistics. For all analyses, the IBM SPSS 20.0 statistics software package was used. To gain information on facilitators and barriers in conducting the intervention and the context, the occupational health professionals were surveyed by means of semi-structured interviews. We schematically sorted the content of the interviews, resulting in a list that was discussed among the research team before a final list was made.

## Results

### Recruitment of the OHS and occupational health professionals

In 2011, the Dutch Health & Safety Institute in the Construction Industry (Arbouw) assigned the implementation of the job-specific WHS to the largest OHS in the Netherlands. Among other OHSs, this OHS was contracted for conducting occupational health care activities for construction workers. Arbouw requested that the OHS have three of their geographically widespread sites throughout the Netherlands participate in performing the job-specific WHS. The OHS assigned a medical´s assistant, an ergonomist and an OP to each department.

### Reach

**Figure 2:**
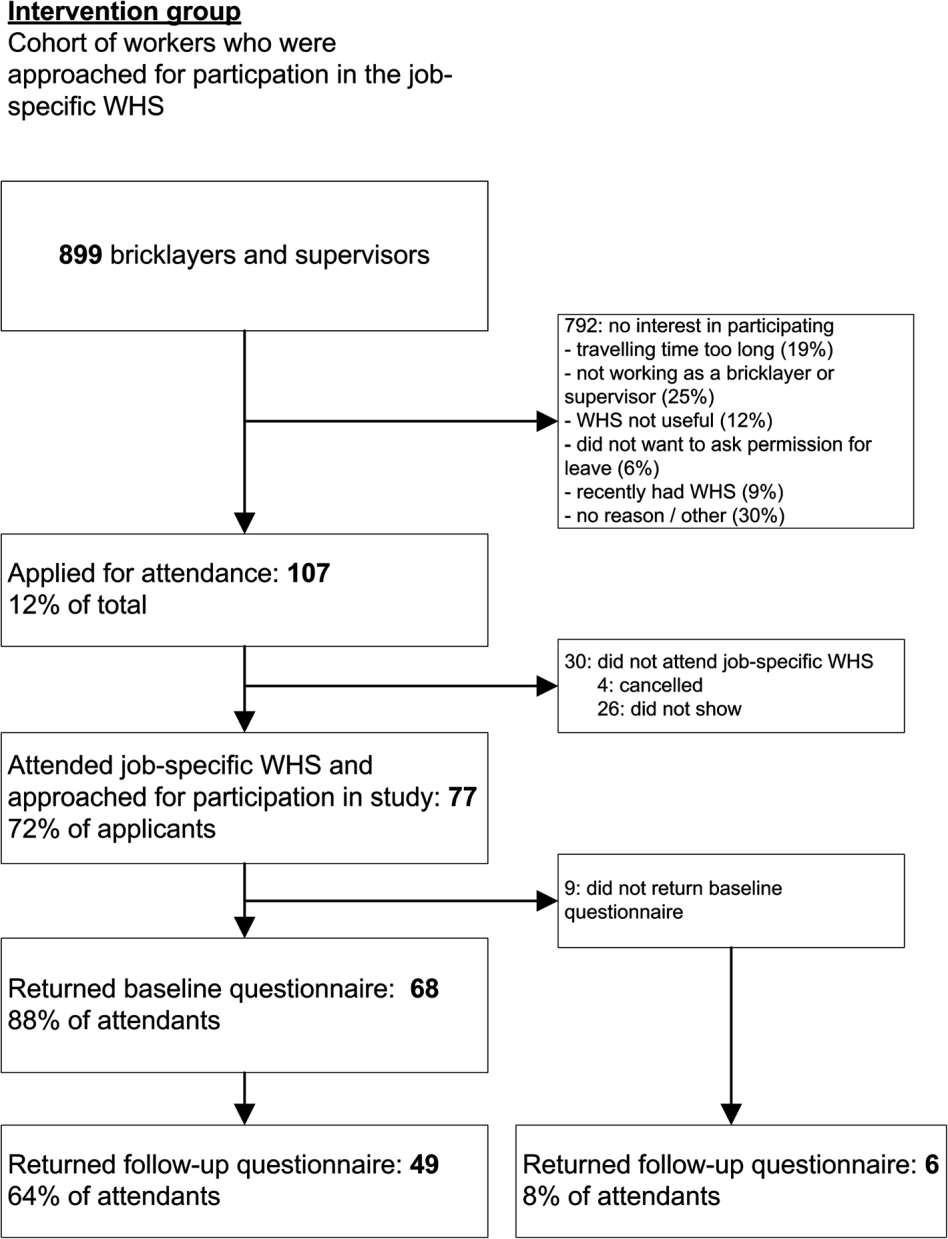
Flowchart of the participants.

Of the 899 bricklayers and supervisors who were invited for job-specific WHS, a total of 107 (12%) made an appointment at the OHS for their job-specific WHS. A total of 792 workers were not interested in participating. Nearly 30% of the population did not give a reason for their non-interest. The most frequently mentioned reason for non-interest was ‘not employed as a bricklayer or supervisor’ (25%), while 19% reported that the travelling time to the OHS was too long (Figure 2).

Next, 77 workers (72% of the applicants) attended their job-specific WHS, comprising 33 bricklayers and 44 supervisors. Among the 30 persons who did not attend their job-specific WHS, 26 did not show up and 4 workers cancelled their appointment. The total reach was 9% (77/899).

### Fidelity

The total performance score ranged between 38% and 100%, with an average score of 67% (Table 3). The most frequent scores were 63% (for 24 workers) and 75% (for 25 workers). For 81% (62/77) of the workers, the performance score was above 55%. At one department, the performance was substantially lower than at the other two departments, as indicated by the number of workers for which a performance score below 55% was obtained (35% compared to 4% and 13%, respectively). An overview of the scores for the eight performance indicators is presented in Table 2. The performance score is lowest for the indicators ‘correct processing of the questionnaire data by medical assistant and OP (sufficient for 16% of the workers) and ‘Signs to intervene were correctly determined based on summary of results’ (sufficient for 16% of the workers).

**Table 3:** Scores for the process evaluation components

**Process evaluation component**	**Outcome**
**Reach**	9% (relative frequency: 77/899)
**Fidelity**	67% (range 38-100%)
**Dose delivered**	Written advice: 92%
	Carrying out follow-up appointments: 63%
**Dose received**	Intention to carry out advice at baseline: 68%
	Remembrance of advice at follow-up: 49%
**Increase in knowledge on health status (scale 0–10)**	
WHS in its entirety	7.0 (SD 1.7) (substantial)
Physical performance tests	6.9 (SD 1.8) (substantial)
Counselling by the OP	6.8 (SD 1.8) (substantial)
**Increase in knowledge on own work ability (scale 0–10)**	
WHS in its entirety	5.9 (SD 2.5) (substantial)
Physical performance tests	6.1 (SD 2.4) (substantial)
Counselling by the OP	6.2 (SD 2.5) (substantial)
**Satisfaction (scale 0–10)**	
WHS in its entirety	7.5 (SD 1.7) (good)
Questionnaire	7.5 (SD 1.3) (good)
Physical performance tests	7.4 (SD 1.6) (sufficient)
Counselling by the OP	7.3 (SD 1.7) (sufficient)
**Perceived (future) effect (scale 0–10)**	
On health status	6.3 (SD 2.6) (sufficient)
On work ability	5.2 (SD 3.0) (limited)

### Dose delivered

All of the workers who attended their job-specific WHS completed all of its components. The OP provided written advice to 92% (65/71) of the workers. The OP planned a follow-up appointment for 10% (8/77) of the workers. For five of these workers (63%, 5/8), the follow-up appointment was undertaken.

### Dose received

Of the 59 workers who reported at baseline to have received one or more recommendations to undertake a preventive action, 18 workers (31%) did not have the intention to follow all of the recommendations that they had been given, compared to 41 who did (69%). A total of 49 workers completed both the baseline and the follow-up questionnaire. Among these 49 workers, a total of 13 workers (27%, 13/49) did not report that they had received any advice on preventive actions at baseline. At follow-up, three months later, 20 workers (41%, 19/49) reported to have undertaken one or more of the preventive actions based on the recommendations that they received, compared to 16 workers (33%, 16/49) who reported that they had not undertaken the advised preventive actions.

Among the 49 workers who completed both the baseline and the follow-up questionnaire, a total of 113 recommendations were reported at baseline and 55 were reported again at the follow-up (49%). This result indicated that 51% (58/113) of the recommendations given by the OP, were not remembered by the workers three months later.

### Programme implementation

The mean score on the components of reach (9%), dose delivered (92%, 63%), dose received (68%, 49%) and fidelity (67%) reflects programme implementation and was found to be 58%.

### Knowledge on own health status and work ability due to attendance of the WHS

Workers were asked how they graded the increase in knowledge about their health status after their health surveillance (Table 3). When asked about the health surveillance in its entirety, two workers indicated that their knowledge of their health status was not increased. A total of 9 workers (12%, 9/73) reported that their knowledge was slightly increased, 36% rated the increase in their knowledge as substantial, and 44% (32/73) noted that they had much more knowledge about their health status as a result of their attendance of the WHS. The contribution of the physical performance tests and the counselling by the OP was rated as good by 42% (30/71) of the workers.

When asked about the effect of the WHS in its entirety with regard to their knowledge of their work ability, most (70%, 51/73) of the workers graded the increase in their knowledge to be little to sufficient. On average, the contribution of the physical performance tests and the counselling of the OP were rated to be sufficient.

### Satisfaction

The workers rated their satisfaction with the job-specific WHS in its entirety and the job-specific questionnaire to be good (Table 3). Their satisfaction with the physical performance tests and counselling by the OP was found to be sufficient.

### Perceived effect

The workers who undertook preventive actions at follow-up, were asked about how they perceived the (future) effect of their actions on their health and work ability. Among the workers for whom this information was available (n=43), the perceived (future) effect on health status was rated as sufficient and the perceived effect on work ability was rated as limited.

### Facilitators and barriers in performing the job-specific WHS

The occupational health professionals mainly addressed barriers to performing the job-specific WHS. They found that the longer duration of the job-specific WHS and the further travel distance to the OHS that performed the intervention might have affected *reach*. Furthermore, due to collective labour agreements, the construction workers are able to spend a specific number of hours yearly for medical purposes, such as attending a health surveillance programme. The workers might have saved this time off for other medical issues that have arisen during the year rather than use them in the first half year, which is the time in which the study was performed.

Concerning *fidelity,* the following aspects were mentioned by the occupational health professionals. The OP´s assistants and the OPs indicated that they had little time to pay the required attention to preparing and completing the documents for the job-specific WHS. Furthermore, the OPs noted that protocol-adherence was sometimes difficult. For example, some workers noted health issues that were not related to the signs. The OPs indicated that they found it difficult to counsel the workers using the intervention protocol. They stated that the protocol contained recommendations, which could not be discussed with the workers. Furthermore, the OPs felt that they needed more knowledge on some of the preventive advises to counsel the workers effectively and indicated that they needed more detailed training on the topic and more practical knowledge. However, the OPs perceived the job-specific questionnaire to be a facilitating factor for detecting the signs of impaired health compared to the general questionnaire, and thereby enhancing decision making and counselling.

The following factors possibly influenced the *dose delivered* according to the OPs. Follow-up appointments were difficult to schedule with the workers, as they were not always willing to come back and take more time off. Furthermore, work place visits were not easy to plan due to the fact that some workers are at a specific construction site for short periods of time and under specific circumstances. The OPs further mentioned that appointments at the workers’ workplace are actually only feasible when the OP is on good terms with the employer and the direction facilitates occupational health care activities, such as workplace visits. However, the explicit advice of the ergonomist to the OP facilitated the opportunity to discuss the advantages or necessity of a workplace visit.

Regarding factors that possibly influenced the *satisfaction* of the workers with the job-specific WHS, the occupational professionals mentioned that some of the workers had to wait for long periods of time between the components of the job-specific WHS. Additionally, especially for the bricklayers, the physical performance test at the end of a working day was quite an effort after a full day of work. However, most of the workers appreciated the comprehensiveness of the job-specific WHS and found it important to participate in a research project.

Regarding the *increase in knowledge* of the workers due to attendance of the job-specific WHS, the occupational health professionals found it difficult to indicate factors related to whether or not the workers had increased knowledge of their health status. Nevertheless, the professionals had the impression that most of the workers were interested in their health status and in the results of the tests and measurements.

### The context of the intervention

Based on the interviews with the occupational health professionals and our own observations during the study, the research team considered the effect of the economic recession in 2011 and 2012 to be a main factor that might have affected both the OHS and the individual workers. Many construction workers had lost their jobs or found themselves in uncertain circumstances. Their willingness to take time off, travel a substantial distance and attend a health surveillance programme that lasts several hours, may have been reduced and could have affected the attendance rate. Additionally, the participating OHS was affected by the recession and had undergone an internal reorganisation with job losses in the preparatory phase of the study. One OP who was originally assigned to participate in the study was replaced by another. This OP did not participate in the half-day training course and was instructed individually, possibly affecting fidelity.

## Discussion

The aim of the present study was to evaluate the process of implementing a job-specific WHS in occupational health care for construction workers in the Netherlands. The results of this process evaluation showed that programme implementation was acceptable. The process elements that affected programme implementation the most, were reach (9%), limited protocol adherence (67%) and limited engagement of the workers with the intervention (55%). The job-specific WHS in its entirety provided the workers with a substantial increase in knowledge related to their health status and on their work ability. Satisfaction with the job-specific WHS and its components was found to be sufficient to good. The workers who had undertaken preventive actions perceived the effect on their (future) health and work ability to be sufficient.

### Strengths and limitations

With this process evaluation we aimed at providing insight into the so-called ‘black box’, to evaluate the job-specific WHS programme and to determine what factors could have affected the outcome. The present study is the first to describe in detail the process of performing WHS for construction workers. We used the practical framework of Linnan and Steckler [18] to develop an evaluation plan. However, several process issues remain unknown. For example, the effectiveness of the recruitment of the sites of the OHS is unknown, as this recruitment was performed by the OHS itself before the actual design of the study. Furthermore, we only considered the behaviour of the occupational health professionals and the workers to be relevant actors, but according to the occupational health professionals participating in the present study, the attitudes of employers and co-workers towards health surveillance are also likely to have an effect on the behaviour and attitude of the workers. Furthermore, we examined the degree to which the follow-up appointments were conducted (as an aspect of the dose delivered), but based on our data we cannot report whether conducting follow-up appointments was correct or incorrect and whether it was due to the OP, the worker, other external factors (such as the employer) or a combination of these elements. To increase the quality of the design and the content of the process evaluation, a step-by-step process-evaluation plan in which the social systems surrounding the workers are also captured, might be a solution [19].

In the framework provided by Linnan and Steckler are recruitment and reach among the variables of interest. Although we documented reasons for non-participation, the conclusions we can draw regarding recruitment and reach are limited based on that information. Therefore, we recommend to put more effort in capturing information on the perceived barriers and facilitators for attending the intervention among both attendants and non-attendants. Questions that could shed light on the low reach, are for example: did the workers read and understand the information provided to them on the job-specific WHS? Did they understand the added value of participating? Was it clear to them how they could sign up for attendance? Although the percentage of workers reached, provides information, interpretation is limited without the underlying causes and potential areas for improvement.

One of our process evaluation components was the workers’ perceived effect on future health and work ability, as the job-specific WHS was designed to improve future health and work ability. By including that measure, we aimed at providing insight in the perceptions of the workers regarding the future effects of the specific WHS. Although this measure is not mentioned by Linnan and Steckler [18], for our job-specific WHS it can be regarded as an important mediator between intervention and outcome. It is likely that workers who do not expect any improvement in their health or work ability due to their actions, will not start or discontinue their actions, conform the theory of planned behaviour [20].

### Comparison with other studies

As we found in the present study, implementing an intervention and conducting research in the construction industry can have its own specific difficulties. The participation rate of the workers was extremely low (9%). In addition to the factors mentioned as barriers for participation, approximately one third of the population of invited workers consisted of workers who skipped their WHS in the previous year. These workers’ interest in WHS may be lower than the average level of interest. Furthermore, one quarter of the population was not working (anymore) as bricklayers or supervisors at the time of the study, and they were in fact wrongfully invited. Although our participation rate was low, similar problems with recruiting companies and participants were reported by Oude Hengel et al. [14] and Groeneveld et al. [21].

The extent to which our intervention was conducted as designed and the requested protocol was followed was limited. Although four of the eight performance indicators were fulfilled for more than 90% of the workers, this figure was 16% for two important performance indicators. This outcome might be due to the lack of time available to conduct the protocol and the abundance of information for the professionals who carried out the job-specific WHS. The interference of the occupational health care setting and the professionals conducting the intervention with adherence to the intervention protocol and thereby affecting fidelity has also been reported by other authors [13,14].

The total programme implementation of the job-specific WHS was found to be 58%. The level of implementation seems to be related to many programme outcomes [12]. Based on a review of 500 studies, positive results have been obtained with implementation levels at approximately 60% [12]. In our study, total programme implementation is composed of the average score of components that went well (for example the intervention dose delivered) and much less positive (for example reach). As indicated by the scores on fidelity, programme implementation varied between the different sites of the OHS, which is a known issue affecting programme implementation [12].

Plat et al. [22] studied the satisfaction of fire fighters with a (mandatory) job-specific WHS. They found high satisfaction among nearly all of the workers (>95%). Although the construction workers in the present study rated their job-specific WHS more conservatively, the job-specific approach seems to appeal to workers in various occupations.

### Implications

The main question that arises as a result of this process evaluation is whether this job-specific WHS can lead to an improvement of occupational health care for construction workers. From the construction sector’s perspective, the answer is likely to be ‘no’, based on the low reach. To improve the health of the population of bricklayers and supervisors by health surveillance, a higher attendance rate seems to be warranted. From the perspective of the individual worker, the answer seems not to be positive either. Fundamental aspects of effective screening, such as the correct processing of the instruments and tests and correct specifying signs to intervene, were not performed well in the present study. However, these issues might be easily solved by better information and communication technology. Furthermore, a substantial portion of the workers did not intend to act upon the advice they were given. Additionally, nearly half of the workers did not reproduce the advice three months later, indicating that their good intentions were only short-term. Independent from the quality of the preventive actions that were actually performed, the potential advantages of attending WHS for the individual’s health are not yet optimal.

A critical reader might wonder whether there are any positive aspects of job-specific WHS. As found in the present study, the workers indicated that they were satisfied with the job-specific WHS and that it substantially increased their knowledge of their health status. Additionally, the workers who undertook one or more preventive actions, indicated that they perceived the (future) effect of their actions on their health status and work ability to be sufficient. In summary, the workers indicated that to some extent the job-specific WHS achieved what it was intended to achieve.

## Conclusions

This process evaluation showed that the implementation of the job-specific WHS was acceptable. Low reach, limited protocol adherence and modest engagement of the workers with the intervention were the most prominent factors that influenced the intervention process. The workers’ increase in knowledge about their health status and work ability was substantial, and the workers’ satisfaction with the intervention was sufficient to good. The perceived effectiveness of the advised preventive actions on the workers’ health status was sufficient. The workers in the present study perceived the effectiveness of the preventive actions on their work ability to be limited.

## Competing interests

The authors declare that they have no competing interest.

## Authors’ contributions

JB was responsible for data collection, analysis, and drafted the manuscript. All authors conceived and designed the study, read and corrected draft versions of the manuscript and approved the final manuscript. HM, JS and MF-D obtained funding for this study. JS and MF-D were the co-principal investigators. All authors read and approved the final manuscript.

## Authors’ information

JKS and MF-D were the co-principal investigators on this project.

## Pre-publication history

The pre-publication history for this paper can be accessed here:

http://www.biomedcentral.com/1471-2458/13/218/prepub
